# Supplementary material for: Synthesis and Physicochemical Characterization of Gelatine-Based Biodegradable Aerogel-like Composites as Possible Scaffolds for Regenerative Medicine
Source: Int J Mol Sci. 2024 May 3;25(9):5009. doi: 10.3390/ijms25095009 (PMC11084852; doi:10.3390/ijms25095009)
Supplement: Supplementary file 1 [file ijms-25-05009-s001.zip › ijms-2959178-supplementary.pdf]

International Journal of
Molecular Sciences

Supplementary Materials

Synthesis and Physicochemical Characterization of Gelatine-Based Biodegradable Aerogel-like Composites as Possible Scaffolds for Regenerative Medicine

Silvana Alfei *, Paolo Giordani, and Guendalina Zuccari

Department of Pharmacy, University of Genoa, Viale Cembrano, 16148 Genoa, Italy
paolo.giordani@unige.it (P.G.); guendalina.zuccari@unige.it (G.Z.)
* Correspondence: alfei@difar.unige.it; Tel.: +39 010 355 2296 (S.A.)

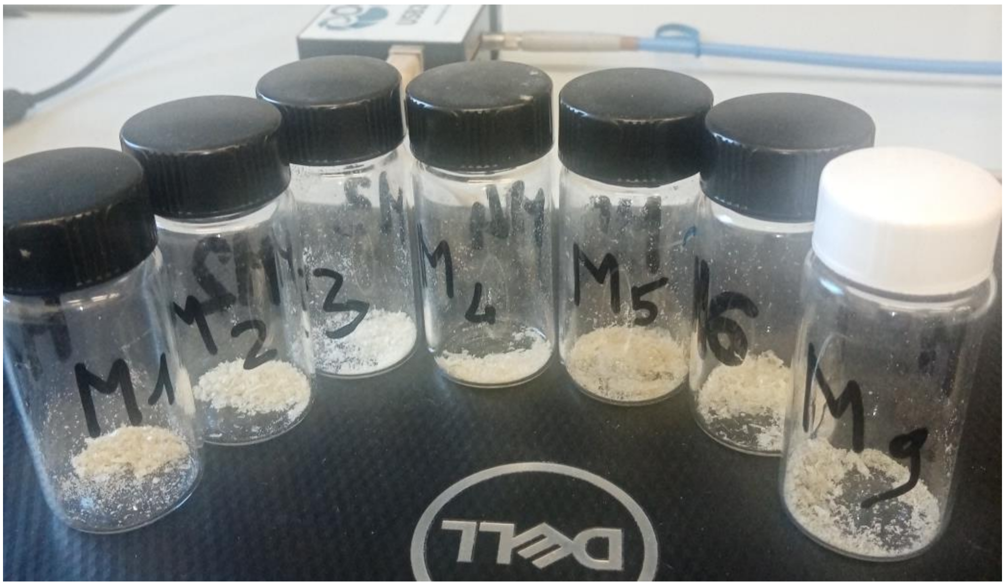

(**a**)

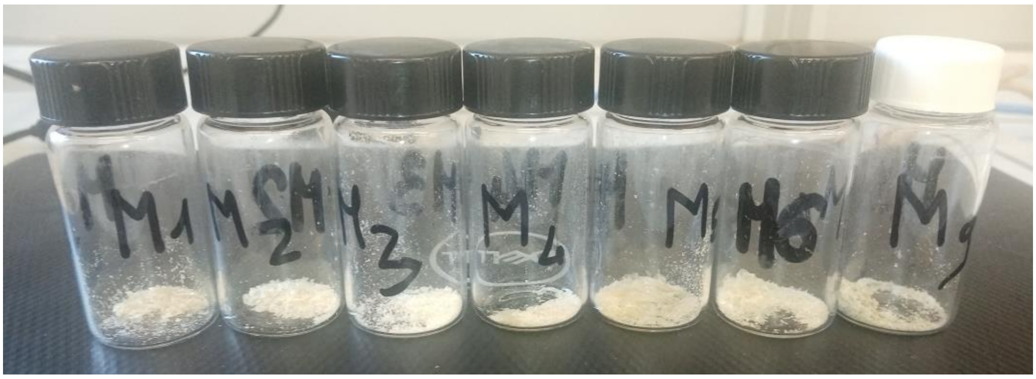

(**b**)

Figure S1. Appearance of aerogel-like samples. A vision from the top (**a**) and a frontal one (**b**).

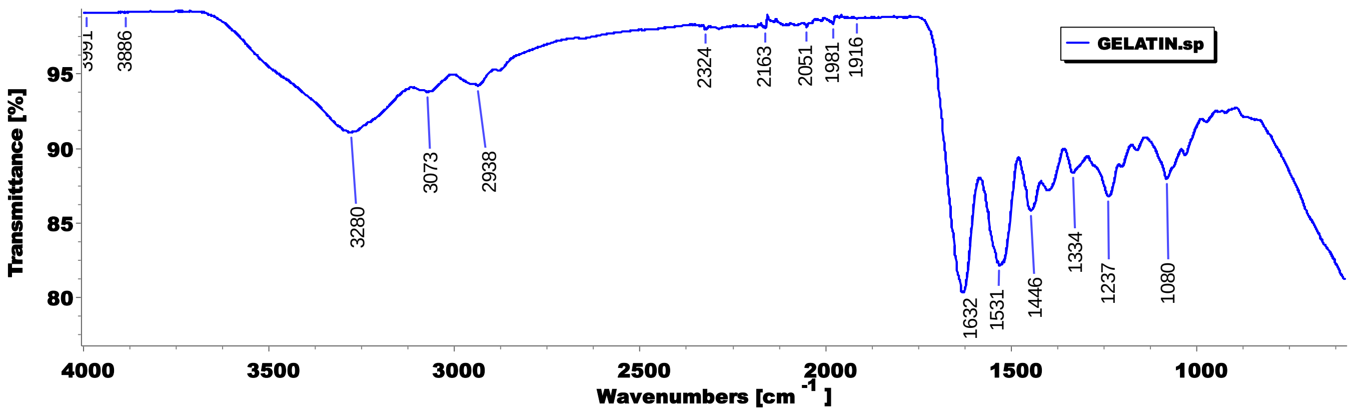

Figure S2. ATR-FTIR spectrum of Gel B.

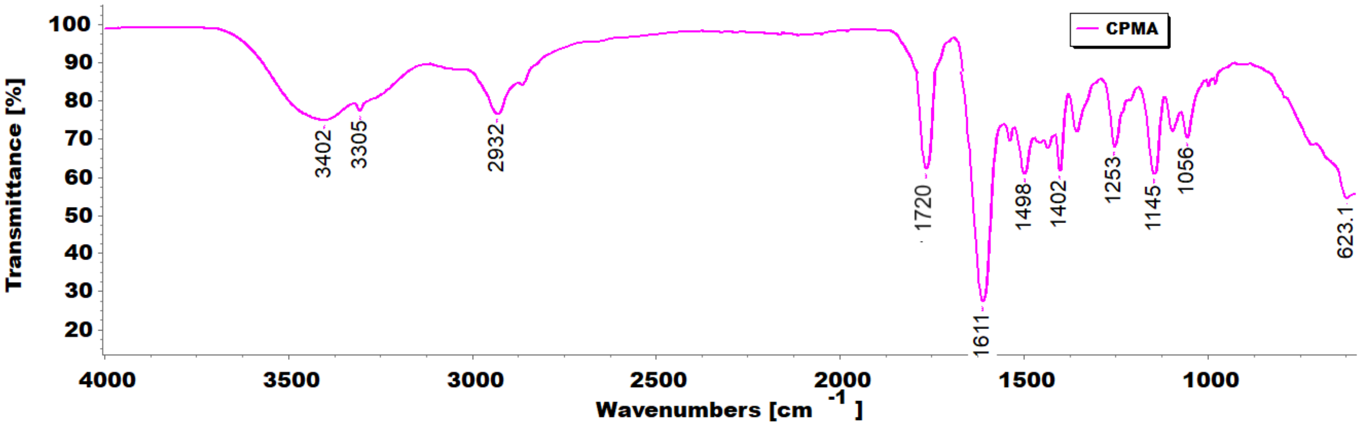

Figure S3. ATR-FTIR spectrum of CPMA/DMAA copolymer.

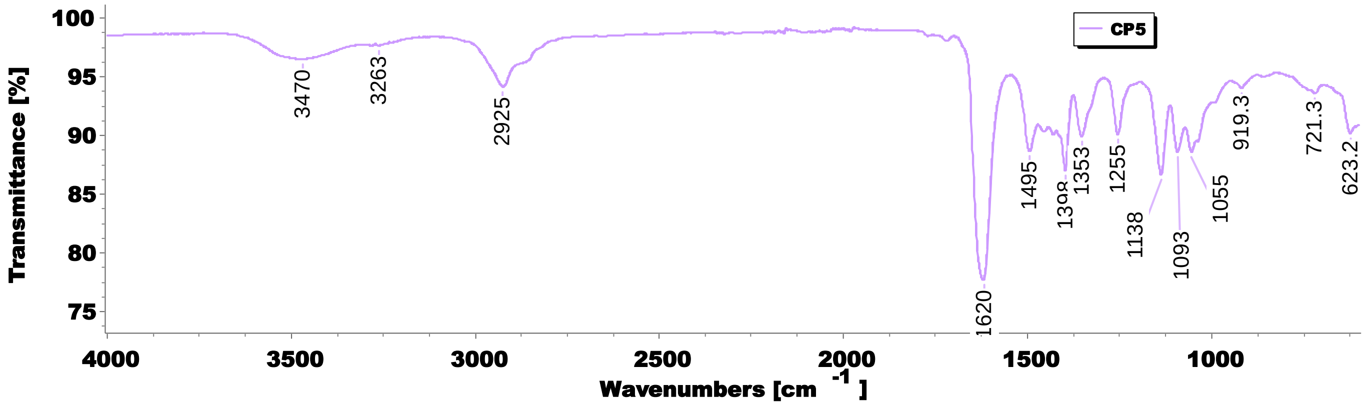

Figure S4. ATR-FTIR spectrum of CP5/DMAA copolymer.

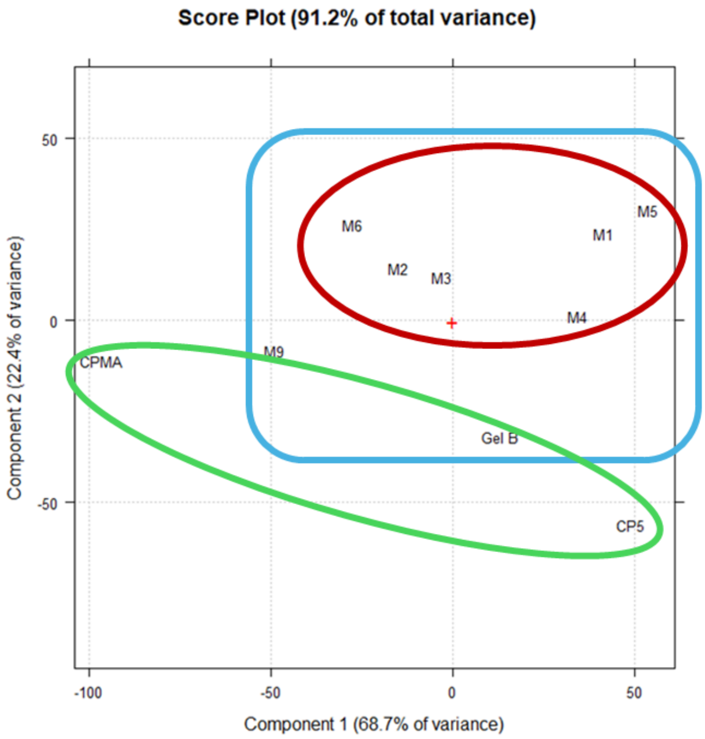

Figure S5. Score plot of PC1 vs. PC2.

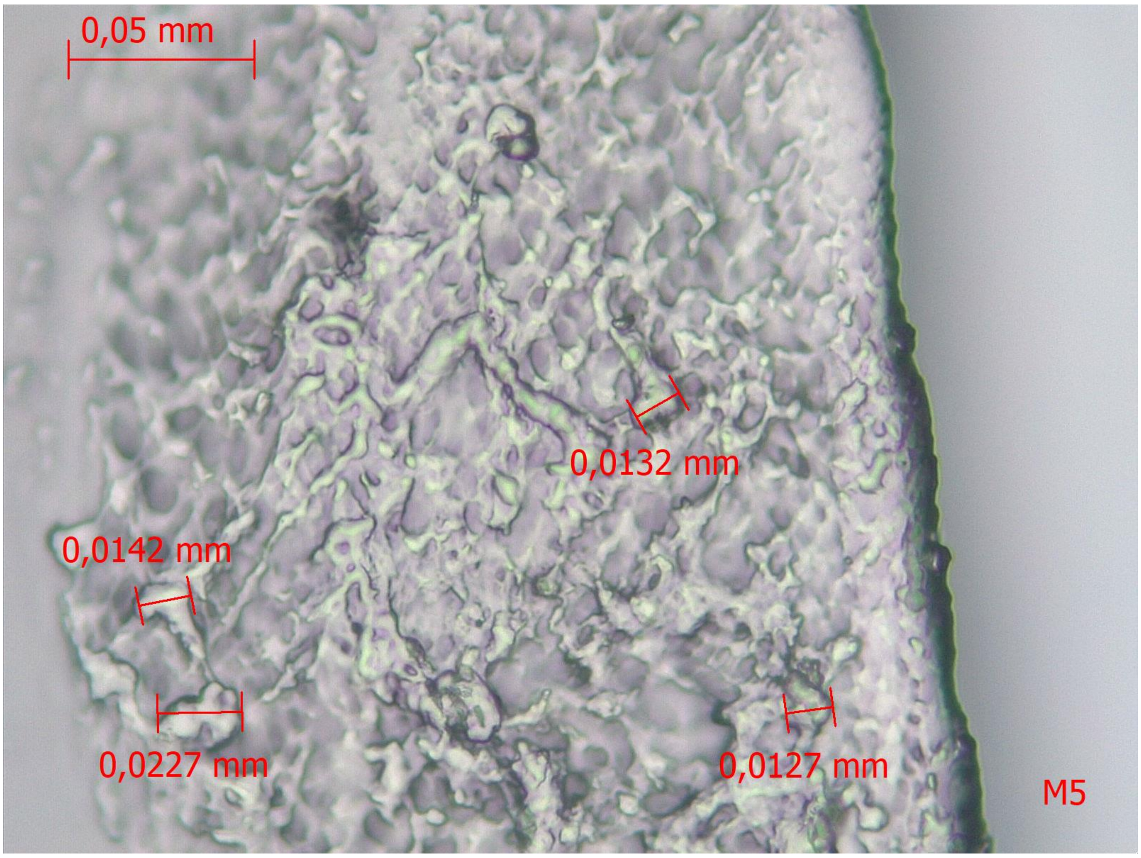

Figure S6. Optical images captured on M5 with a 40 × objective.

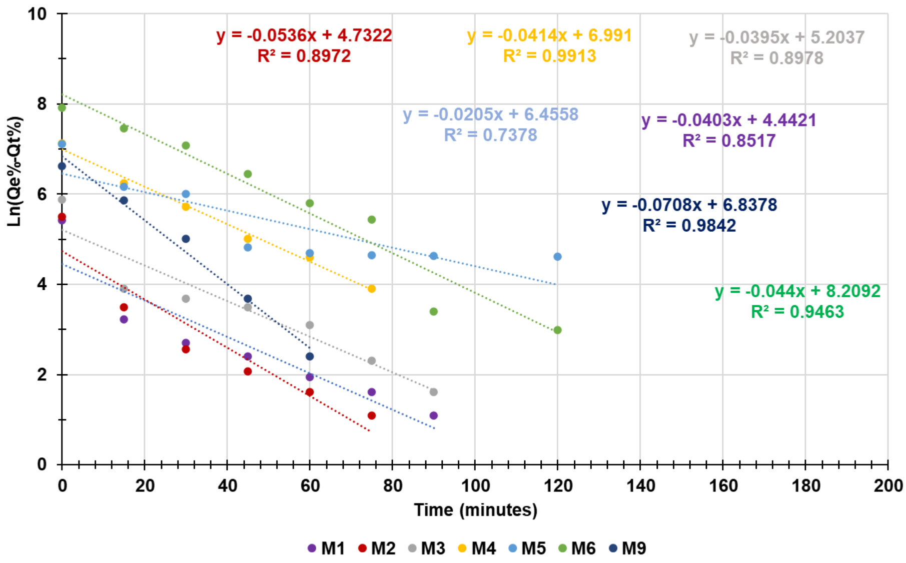

Figure S7. Pseudo first order kinetic model.

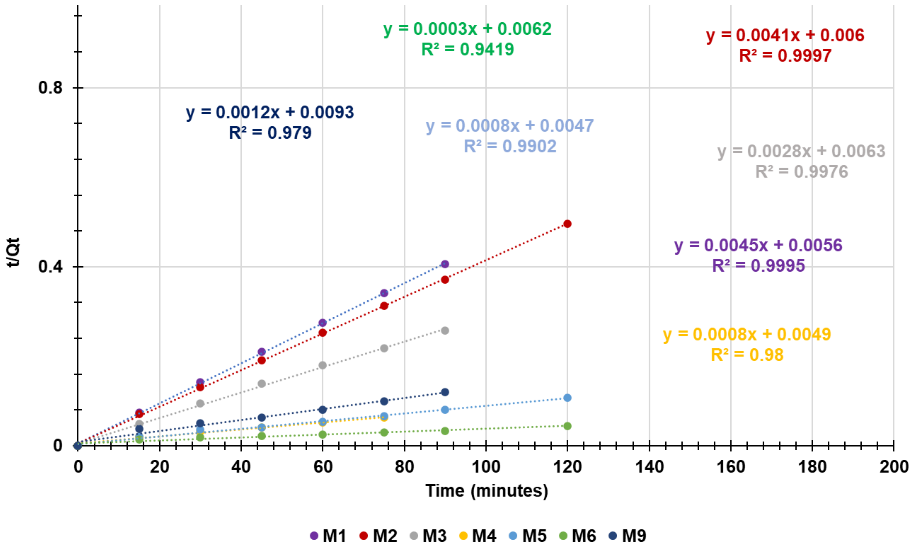

Figure S8. Pseudo second order kinetic model.

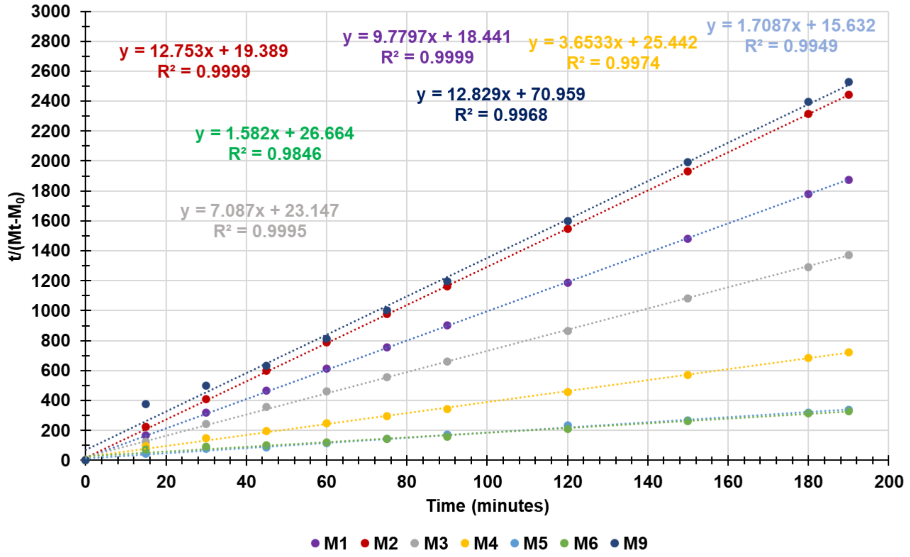

Figure S9. Peleg's kinetic model.

Table S1. Values of coefficients of determinations (R^2) obtained for the kinetic models fitted to swelling experimental data.

	Coefficients of Determination (R^2)		
Kinetic Model	PFO	PSO	Peleg
M1	0.8517	0.9995	**0.9999** *
M2	0.8972	0.9997	**0.9999** *
M3	0.8978	0.9976	**0.9995** *
M4	0.9913	0.9800	**0.9974** *
M5	0.7378	0.9902	**0.9949** *
M6	0.9463	0.9419	**0.9846** *
M9	0.9842	0.9790	**0.9968** *

* Numbers in bold indicate the highest values of R^2 and the mathematical model which best fit the cumulative swelling rate (%) data.

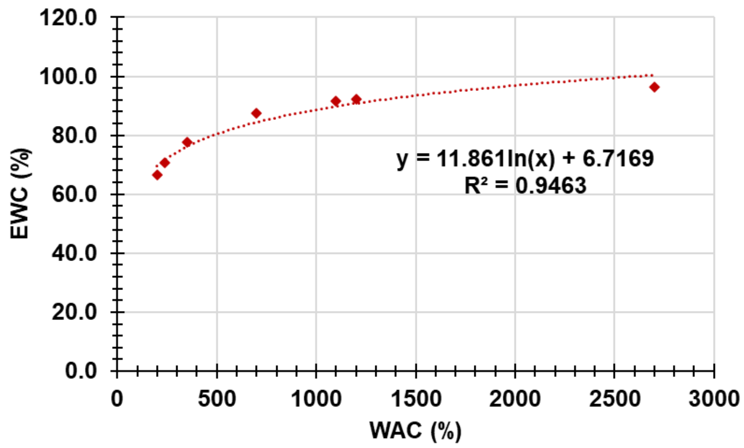

Figure S10. Logarithmic correlation existing between WAC (%) and EWC (%).

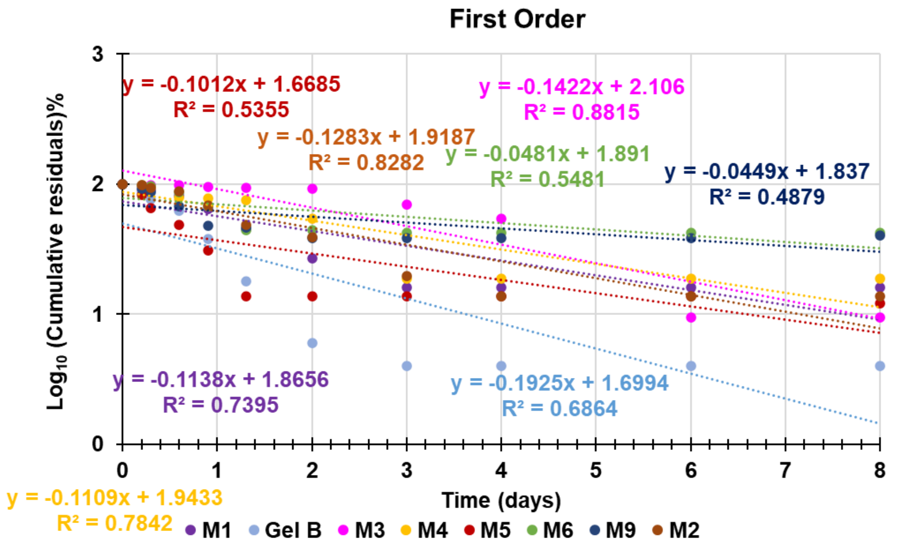

Figure S11. First order kinetic model.

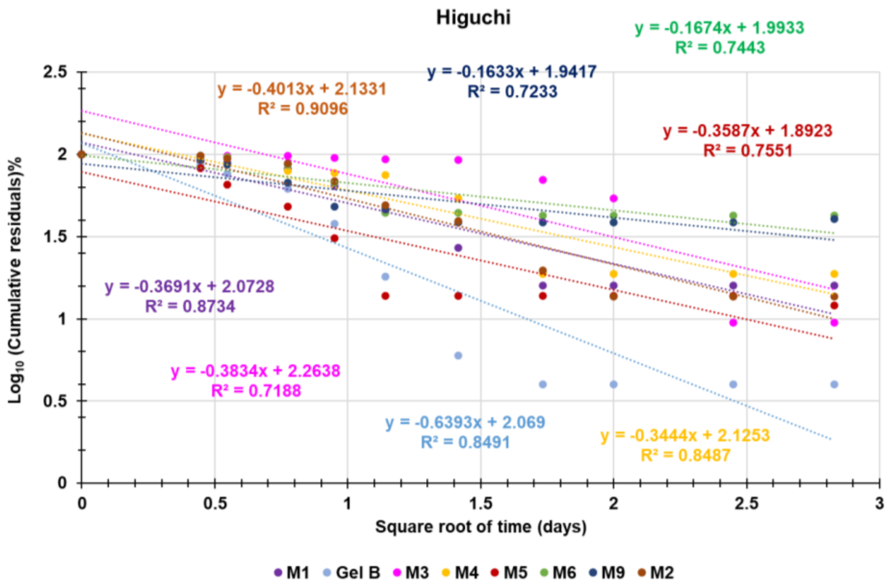

Figure S12. Higuchi kinetic model.

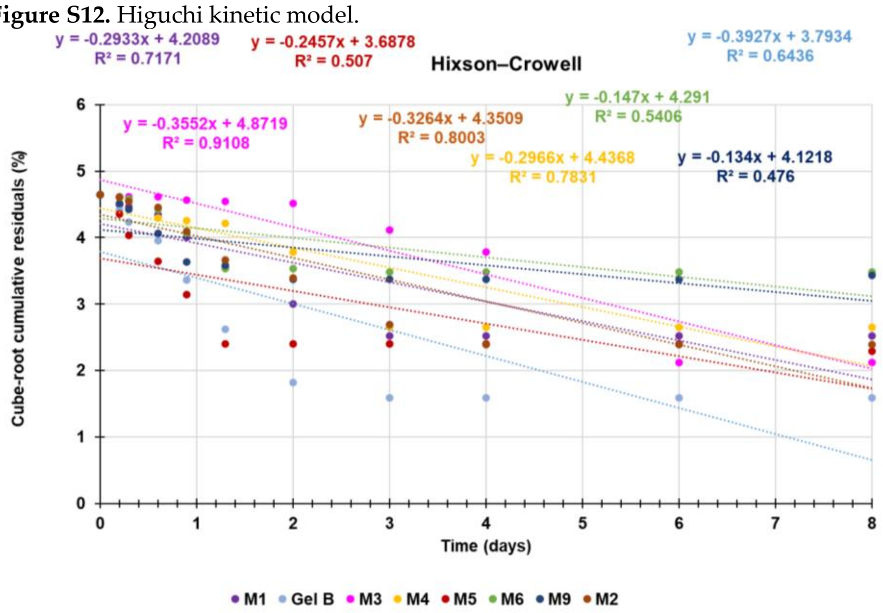

Figure S13. Hixson-Crowell kinetic model.

Table S2. Values of the coefficients of determination (R^2) for all the kinetic models fitted to degradation experimental data.

Kinetic Model	First-order	PSO	Higuchi	Korsmeyer-Peppas	Hixson–Crowell
**R^2 of Gel B**	0.6864	**0.9860** *	0.8491	0.8308	0.6436
**R^2 of M1**	0.7395	**0.9879** *	0.8734	0.8963	0.7171
**R^2 of M2**	0.8282	**0.9754** *	0.9096	0.8753	0.8003
**R^2 of M3**	0.8815	0.8363	0.7188	**0.9260** *	0.9108
**R^2 of M4**	0.7842	**0.9771** *	0.8487	0.8982	0.7831
**R^2 of M5**	0.5355	**0.9920** *	0.7551	0.7376	0.5070
**R^2 of M6**	0.5481	**0.9985** *	0.7443	0.7850	0.5406
**R^2 of M8**	0.4879	**0.9975** *	0.7233	0.7381	0.4760

* Numbers in bold indicate the highest values of R^2 and the mathematical model which best fit the cumulative mass loss (%) data.

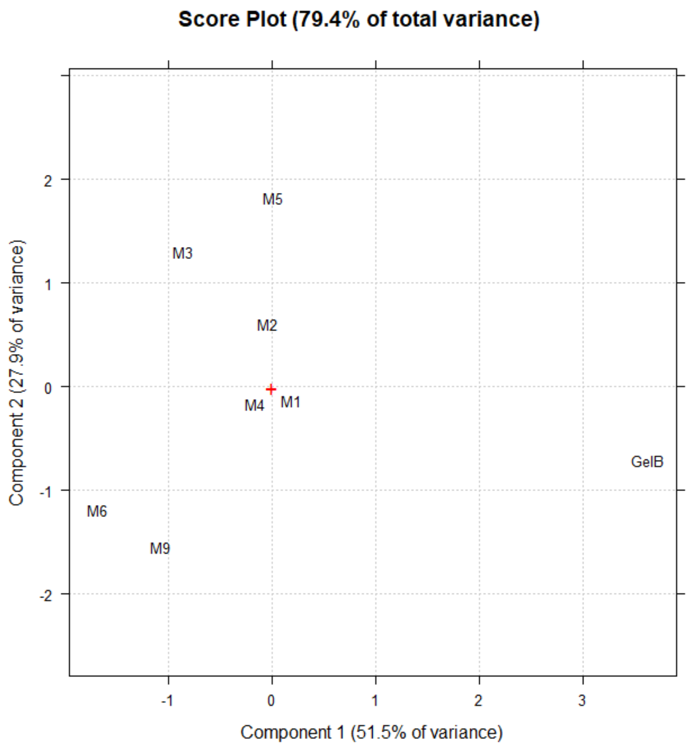

Figure S14. Results of PCA as score plot of PC1 vs PC2.

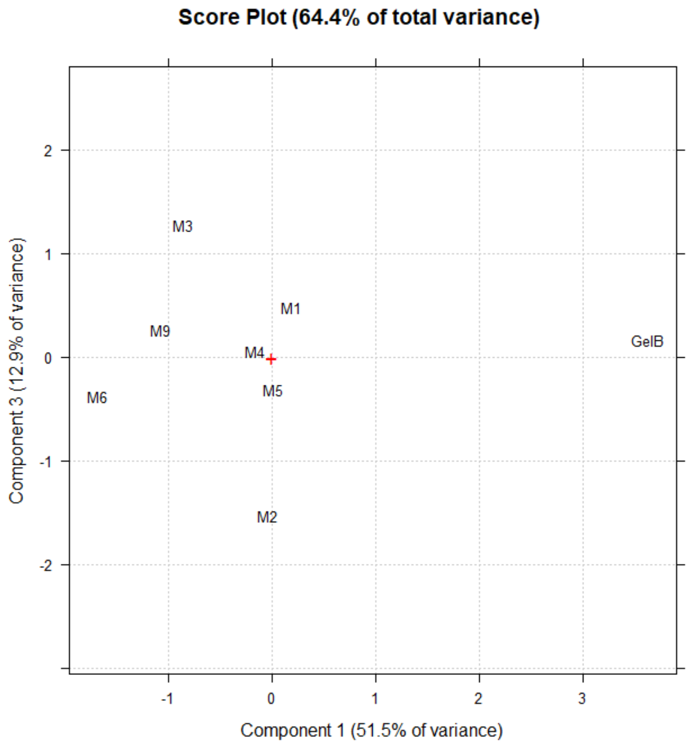

Figure S15. Results of PCA as score plot of PC1 vs PC3.

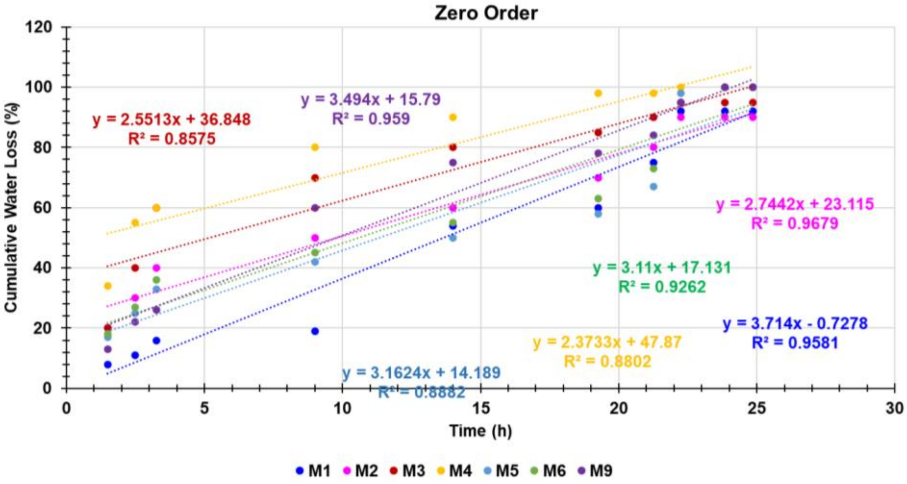

Figure S16. Zero order kinetic model.

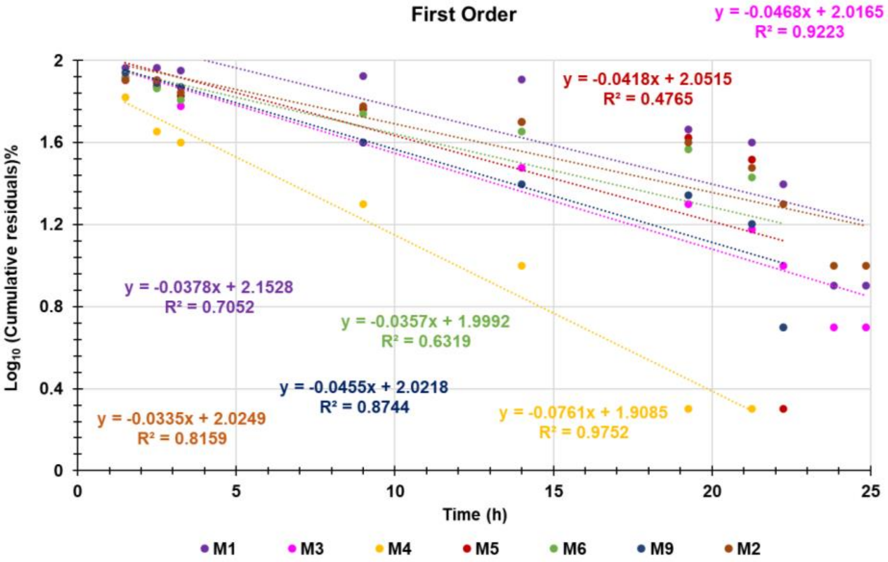

Figure S17. First order kinetic model.

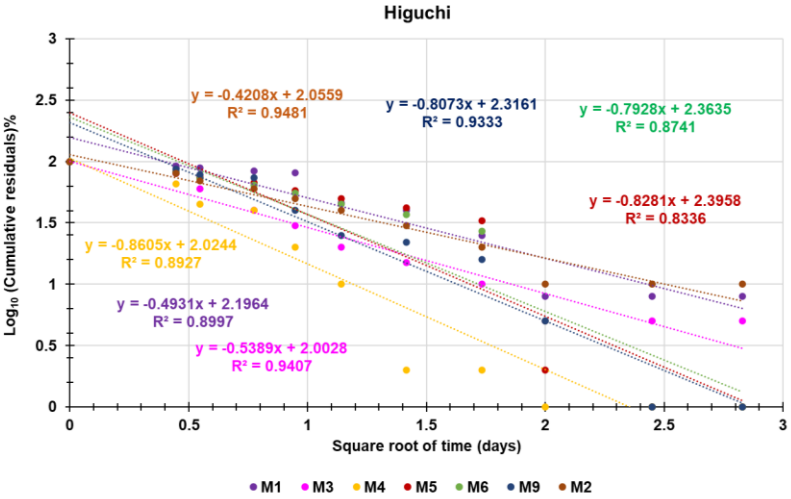

Figure S18. Higuchi kinetic model.

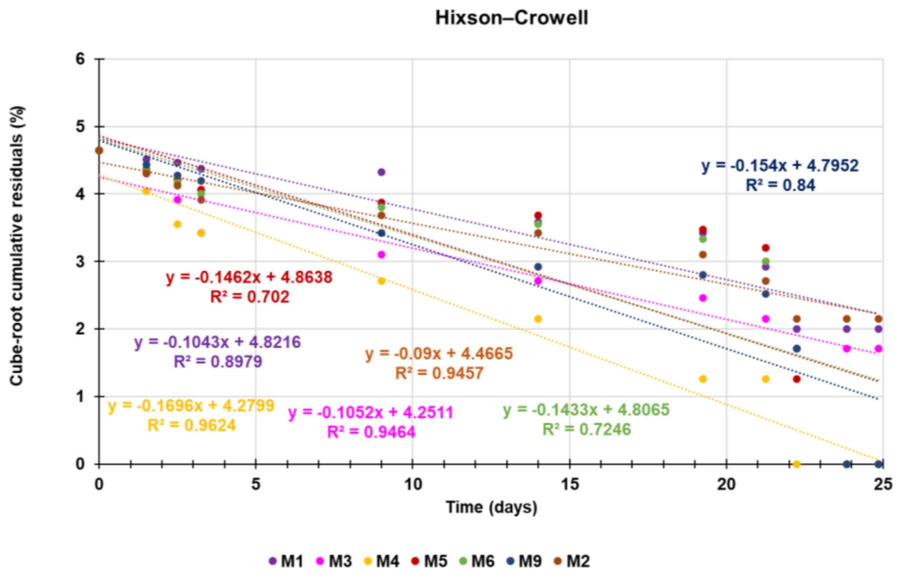

Figure S19. Hixson-Crowell kinetic model.

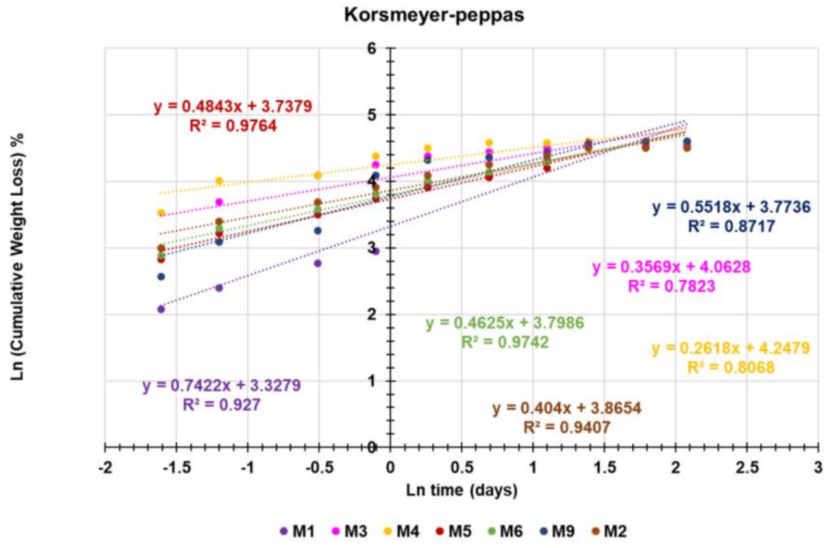

Figure S20. Korsmeyer-Peppas kinetic model.

Table S3. Values of the coefficients of determination (R^2) for all the kinetic models fitted to water loss experimental data.

Kinetic Model	Zero order	First-order	Higuchi	Korsmeyer-Peppas	Hixson–Crowell
R^2 of M1	**0.9581** *	0.7052	0.8997	0.9270	0.8979
R^2 of M2	**0.9679** *	0.8159	0.9481	0.9407	0.9457
R^2 of M3	0.8575	0.9223	0.9407	0.7823	**0.9464** *
R^2 of M4	0.8802	**0.9752** *	0.8927	0.8068	0.9624
R^2 of M5	0.8882	0.4765	0.8336	**0.9764** *	0.7020
R^2 of M6	0.9262	0.6319	0.8741	**0.9742** *	0.7246
R^2 of M8	0.9590	0.8744	0.9333	0.8717	0.8400

* Numbers in bold indicate the highest values of R^2 and the mathematical model which best fit the cumulative mass loss (%) data.

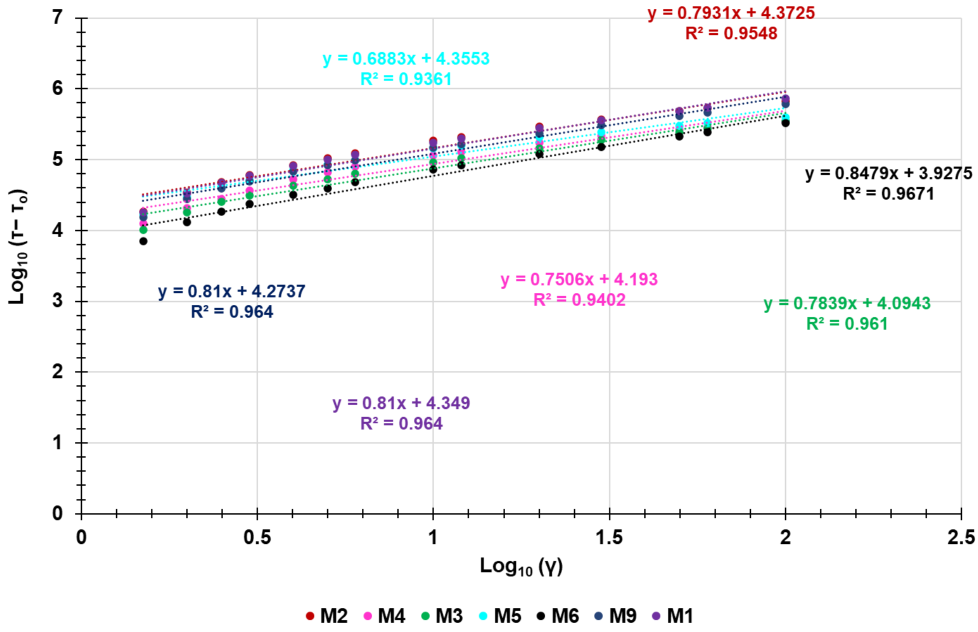

Figure S21. Hershel–Buckley rheological models.

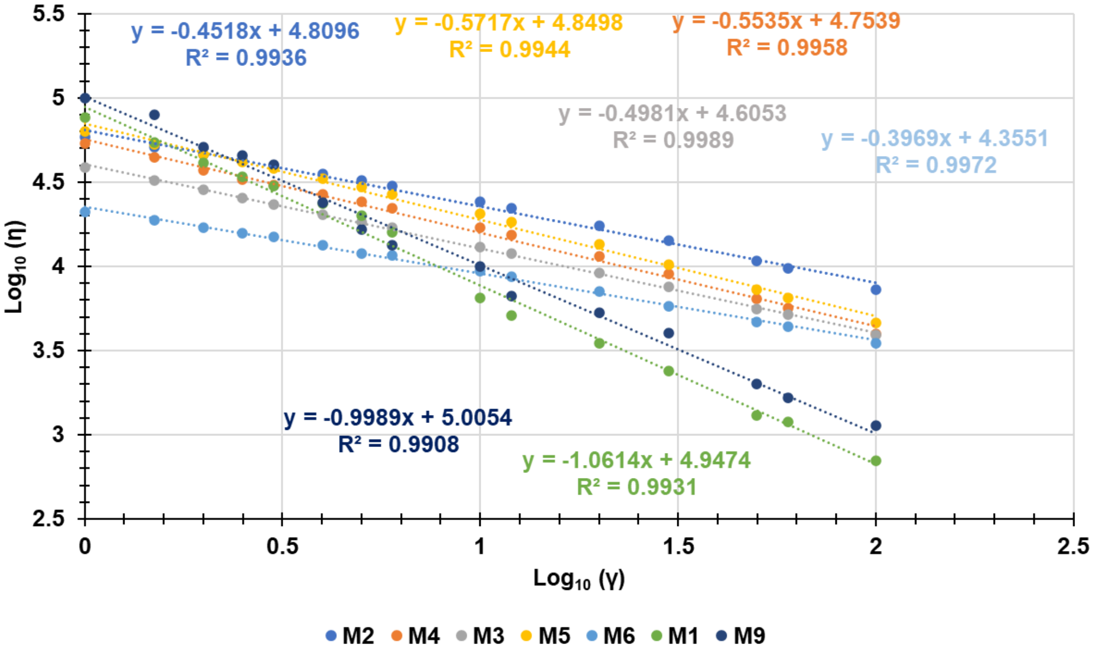

Figure S22. Cross rheological models using equation (12) in the main text.

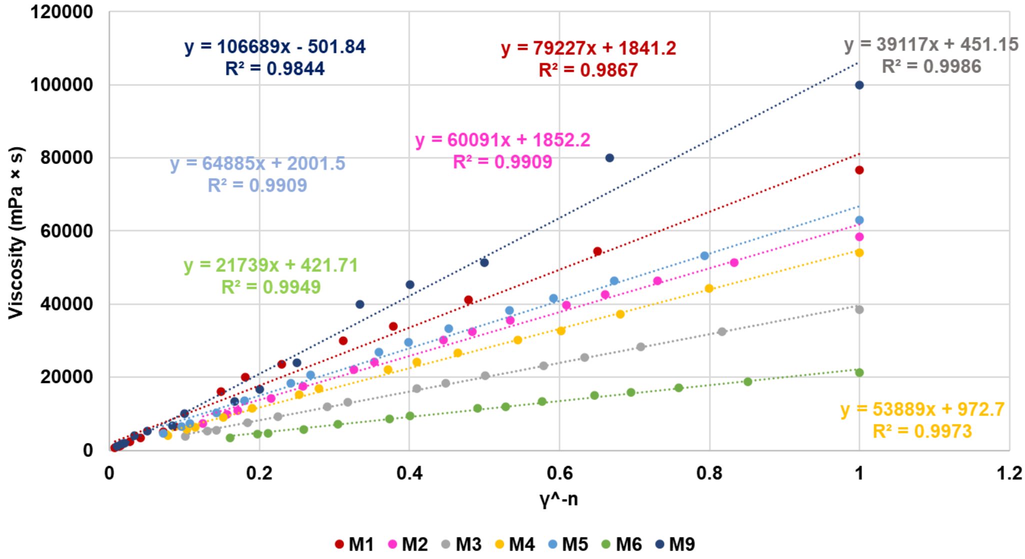

Figure S23. Cross rheological models using equation (13) in the main text.

Table S4. Data obtained by fitting the rheological data to the Cross mathematical model using Eq. (12).

Sample	Equation	R^2	Slope (n)	Intercept ($Log\ (\eta_o/\alpha)$)	α *
M1	y = -1.0614x + 4.9474	0.9931	1.0614	4.9474	20.66
M2	y = -0.4518x + 4.8096	0.9936	0.4518	4.8096	28.70
M3	y =- 0.4981x + 4.6053	0.9989	0.4981	4.6053	11.07
M4	y = -0.5535x + 4.7539	0.9958	0.5535	4.7539	17.30
M5	y = -0.5717x + 4.8498	0.9944	0.5717	4.8498	28.28
M6	y = -0.3969x + 4.3551	0.9972	0.3969	4.3551	18.42
M9	y = -0.9989x + 5.0054	0.9908	0.9989	5.0054	4.9

* Obtained using intercepts and values of η_o reported in Table S5.

Table S5. Data obtained by fitting the rheological data to the Cross mathematical model using Eq. (13).

Sample	Equation	R^2	Intercept (η_∞, mPa × s)	Slope (η_o/α)	η_o (mPa × s)	α **
M1	y = 79,227x + 1,841.2	0.9867	1,841	79,227	1,841,000	23.24
M2	y = 60,091x + 1,852.6	0.9909	1,853	60,091	1,853,000	30.83
M3	y = 39,117x + 451.2	0.9986	451	39,117	451,000	11.53
M4	y = 53,889x + 972.7	0.9973	973	53,889	973,000	18.05
M5	y = 64,885x + 2,001.5	0.9909	2,002	64,885	2,002,000	30.85
M6	y = 21,739x + 421.7	0.9949	422	21,739	422,000	19.41
M9	y = 106,689x – 501.8	0.9844	502	106,689	502,000	4.7

* η_o calculated as 1000 × η_∞; ** obtained using slopes and values of η_o reported in the Table.
